# Promoting MedlinePlus utilization in a federally qualified health center using a multimodal approach

**DOI:** 10.5195/jmla.2018.216

**Published:** 2018-07-01

**Authors:** Mechelle Sanders, Kate Bringley, Marie Thomas, Michele Boyd, Subrina Farah, Kevin Fiscella

**Affiliations:** Department of Family Medicine Research Programs and Department of Public Health Sciences, University of Rochester, 1381 South Avenue, Rochester, NY 14620; Woodward Health Center, Anthony L. Jordan Health Center, 480 Genesee Street, Rochester, NY 14611; Department of Family Medicine Research Programs, University of Rochester, 1381 South Avenue, Rochester, NY 14620; Department of Family Medicine Research Programs, University of Rochester, 1381 South Avenue, Rochester, NY 14620; Department of Family Medicine Research Programs, University of Rochester, 1381 South Avenue, Rochester, NY 14620; Department of Family Medicine Research Programs and Department of Public Health Sciences, University of Rochester, 1381 South Avenue, Rochester, NY 14620

## Abstract

**Background:**

Most patients want more health information than their clinicians provide during office visits. Written information can complement information that is provided verbally, yet most primary care practices, including federally qualified health centers, have not implemented systematic programs to ensure that patients receive understandable, relevant, and accurate health information at the point of care. MedlinePlus in particular is underutilized.

**Case Presentation:**

The authors conducted a multimodal intervention to promote the use of MedlinePlus at a federally qualified health center. We provided MedlinePlus training to clinicians and patients through group and one-on-one trainings and multimedia promotion. We administered pre- and post-intervention surveys to patients, clinicians, and nurses to assess changes in the use and recognition of MedlinePlus at the point of care. We used quantitative and qualitative data to understand the impact of the intervention. A National Library of Medicine grant provided resources that supported equipment and staff. Group training improved use of MedlinePlus by clinicians and staff. One-on-one training was most effective for patients, particularly when it was integrated into the work-flow.

**Conclusions:**

A multimodal approach can promote use of MedlinePlus among community health center patients. However, the process is labor- and resource-intensive and requires careful attention to work flow and leveraging of brief opportunities.

## BACKGROUND

Inadequately informed decision-making by patients is common in primary care [1–4]. Many patients—particularly those with low health literacy, low education, or limited English proficiency—lack the relevant knowledge about their medications or health conditions that is necessary to engage in informed decision-making or self-management [5–13]. In a study of underserved patients, only half could cite their medication names, indications, and frequency, while only 25% knew their doses and none could report potential side effects [14]. African American patients experience more medication problems that are potentially related to worse access to high-quality health information [15, 16].

Clinicians often misjudge patients as being more informed than patients actually are [17], while patients believe that they understand more than they actually do [18].

MedlinePlus is a high-quality health information website produced by the US National Library of Medicine (NLM) of the National Institutes of Health. Information on the website is continuously updated, scientifically vetted, and free of advertisements and product endorsements [19]. The website covers more than 975 diseases and conditions, drugs, and supplements in addition to providing access to videos and tools. The full website is available in English and Spanish and includes easy-to-read sections written at 5th to 8th grade reading levels. MedlinePlus also offers Connect, an application programming interface that can be integrated with selected electronic health records (EHRs) and portals. The program provides relevant information on diagnoses, medications, and lab tests at the point of care [20].

## STUDY PURPOSE

This case study describes the authors’ experience in promoting the use of MedlinePlus among poor and minority patients and their clinicians in a federally qualified health center (FQHC). Our project had three major goals: (1) improve web access to MedlinePlus for poor or minority patients; (2) improve patients’ knowledge, attitude, and skills related to accessing online health information; and (3) improve clinicians’ use of MedlinePlus at the point of care.

## CASE PRESENTATION

Our project involved collaboration between an academic medical center and a large urban FQHC. FQHCs are independent, nonprofit organizations that serve low-income populations. At this FQHC, 78% of patients live at or below 100% of the federal poverty level. The FQHC comprises 4 major sites that provide patient care throughout the community. The University of Rochester Institutional Review Board reviewed the project and determined it to be exempt from review. An NLM grant (G08 LM011524-01) provided resources that supported equipment and staff. The funders had no role in study design, data collection, analyses, or decision to publish the manuscript.

### Project steering committee

We created a project steering committee consisting of the project director, project coordinator, research assistant, consulting medical school librarian, and health center staff (including the health center’s director of clinical operations, practice managers, and key nursing staff). Using Carman et al.’s [21] framework as a guiding principle, the committee identified three levels (i.e., patients, clinicians, and the organization) for targeting in order to improve use of MedlinePlus. The committee brainstormed various strategies based on behavioral and organizational principles, feasibility, and likely yield. The committee settled on seven strategies of implementation, focusing on our three project goals (Table 1). The committee met regularly throughout the project to oversee implementation, assess progress, problem-solve barriers, and recommend adjustments. The medical school librarian compiled resources, provided advice to the group, compiled curricular materials, assisted in training clinicians and nurses in the use of MedlinePlus, and served as resource for ongoing questions about the use of MedlinePlus.

**Table 1:** Implementation strategies

	Methods	Time frame	Pros	Cons
Goal 1: Improve patients’ access to MedlinePlus	Computer kiosks in waiting room	11/2013–8/2016	Inexpensive and low maintenance	Low patient uptake
Goal 2: Improve patients’ knowledge, attitudes, and skills related to accessing online health information	Computer classes	4/2014–8/2015	In-depth learning opportunity	Very low patient uptake; much staff time needed to recruit patients and host classes
	Diabetes group training sessions	5/2016–8/2016	High patient uptake (100% attendance for 4 out of 6 classes)	Much staff and clinician time needed to recruit patients and host classes
	Waiting room demonstrations and recurring MedlinePlus video loops on waiting room television monitors	6/2014–8/2016	Live demonstrations drew waiting room patients’ interest; 25% of patients who were approached agreed to be trained; video loops could be seen by all patients as they waited for appointments	Much staff and clinician time needed to canvas the waiting rooms and perform the demonstrations
Goal 3: Improve clinicians’ use of MedlinePlus at the point of care	Integrate MedlinePlus Connect into electronic health record (EHR)	NA	NA	Due to incompatibility issues, we were unable to integrate MedlinePlus Connect into the EHR
	Clinician and nurse training on use of MedlinePlus during office visits	3/2014–8/2016	80% of clinicians and nurses attended the training sessions	Requires strong cooperation from clinicians and nurses; time constraints
	Licensed practical nurse (LPN) training on incorporating MedlinePlus into after visit summary (AVS) reviews and nurse visits	1/2014–8/2016	Very few refusals from patients; LPNs reported ease of integration into current care processes	Requires strong cooperation from LPNs; time constraints

### Strategy implementation

#### Goal 1: Improve patients’ access to MedlinePlus

To improve patient access to MedlinePlus, we refurbished the computer kiosks in the waiting rooms and added a bookmark for MedlinePlus. Each health center had one kiosk in its waiting room. Each computer received an upgraded hard drive and operating system, a new keyboard, and a new monitor. Patients could use the kiosks to look up health information on the web before or after their appointments. We created posters and placed them throughout the health center encouraging patients to use the kiosks.

#### Goal 2: Improve patients’ knowledge, attitude, and skills related to accessing online health information

To address basic computer literacy, one of the health center sites piloted weekly classes for adult patients that addressed basic computer skills and how to effectively use MedlinePlus. On the day of the class, up to ten patients were provided a laptop that they could use during the class session. The class topics included basic computer operation, finding and evaluation of online health resources, and navigation of MedlinePlus. We promoted classes via posters and health center staff. We also piloted integration of MedlinePlus training into weekly Diabetes Group visits. Patients used smartphones to access diabetes sections of MedlinePlus. We offered light refreshments and transportation vouchers to incentivize class attendance.

Each of the health centers also piloted waiting room demonstrations of MedlinePlus. A part-time student working at the FQHC approached patients in the waiting rooms and asked if they would be willing to participate in a one-on-one demonstration of MedlinePlus using a laptop or iPad. The sessions typically lasted approximately ten minutes, depending on the patient’s skills. The student also assisted patients who owned smartphones with bookmarking the MedlinePlus website on their phones and signing up for the practice’s EHR patient portal. This strategy gave rise to the creation of MedlinePlus videos shown through video loops on the waiting rooms’ television monitors. The videos encouraged patients to visit MedlinePlus and provided a brief overview of the website.

#### Goal 3: Improve clinicians’ use of MedlinePlus at the point of care

We developed and delivered group trainings for health center clinicians and nurses to improve patient access to target high-quality information at the point of care. During these lunchtime trainings, we reviewed key features of MedlinePlus and demonstrated how to integrate it into patient visits. We hosted the training sessions over lunchtimes to minimize disruptions.

Prior to the delivery of the group training sessions, the steering committee met with the FQHC’s Information Technology Department to try and integrate MedlinePlus in the health centers’ EHR. Unfortunately, the FQHC’s EHR could not be configured to link to MedlinePlus Connect. As a work-around, the MedlinePlus website was bookmarked on all of the desktop computers in the patient exam rooms for quick and easy access.

In addition, we trained nurses working with clinicians on ways to briefly demonstrate MedlinePlus during the after visit summary (AVS) reviews with each patient. The Centers for Medicare & Medicaid Services endorses AVSs, which include a summary of the diagnoses, medication changes, immunizations and testing, referrals, and next visits [22]. Because the nurses spent more time with the patients than the clinicians did during the AVSs, there was an opportunity to review MedlinePlus and assist the patient in using it themselves. The MedlinePlus demonstrations by the nurses augmented clinical information or health promotion strategies that were included in the AVS.

We also purchased iPads and laptops for patients to use in the exam rooms. Clinicians instructed patients to go to the MedlinePlus site using the iPad or laptop and view interactive videos regarding topics such as cancer screening options and management of chronic health conditions

### Measures

We conducted a mixed methods evaluation of the project. Mixed methods integrate quantitative and qualitative data collection approaches to derive real-world contextual understanding and perspectives from multiple vantage points [23]. We used surveys to collect quantitative data and performed observations and reviewed meeting notes as qualitative data to evaluate the impact of implementation of the seven strategies to improve patient and clinician recognition and use of MedlinePlus.

We surveyed patients’ use of MedlinePlus using two samples of patients in the health centers’ waiting rooms prior to their scheduled doctors’ visits. One sample was surveyed before project implementation, and the other sample was surveyed two years after the beginning of project implementation. Health center staff distributed paper surveys to eligible patients upon check-in. Patients were eligible to complete the survey if they had a scheduled appointment, were at least eighteen years of age, and spoke English. The survey included questions regarding frequency of use, interest, and knowledge about MedlinePlus (supplemental Appendix A). The research assistant entered the completed surveys into REDCap, which is a secure, web-based application for electronic collection and management of research and clinical trial data [24].

We also conducted a survey of health center clinicians and nurses (supplemental Appendix B). The survey included questions regarding use of MedlinePlus and recommendation of the website to patients. A staff assistant emailed all clinicians and nursing staff a link to complete the survey online via REDCap [24]. The staff assistant sent a second email request three weeks later for nonresponders.

For the process evaluation, a research assistant coded the notes from steering committee meetings and observational notes recorded throughout the entire study period for emergent qualitative themes. The objective was to understand the barriers and facilitators associated with implementation and uptake of the seven strategies. Descriptive statistics for characteristics of the patients, clinicians, and nursing staff were tabulated. Data were analyzed using STATA version 12.1 (StataCorp, College Station, TX, USA).

### Strategy assessment

#### Goal 1: Improve patients’ access to MedlinePlus

To assess this goal, we compared results from pre-intervention (n=302 patients) and post-intervention (n=302) cross-sectional patient surveys. The demographic characteristics of the patients who were surveyed before and after implementation were similar (supplemental Table 2). In general, respondents were largely female, middle-aged, and minority (African American and Latino) and had low levels of educational attainment.

We found that the use of MedlinePlus increased significantly from 2% before intervention to 6% after intervention (χ^2^(1)=5.24, *p*=0.02) (Table 3). There was no statistically significant difference in the proportion of patients who reported hearing about MedlinePlus before and after intervention. However, our qualitative data revealed that some patients who reported not using MedlinePlus were actually using MedlinePlus but had forgotten the name.

**Table 3:** Patient pre- and post-intervention survey results

	Pre-intervention	Post-intervention	
How OFTEN have you used the Internet to look up information about your health?	(n=295)	(n=301)	NS
Never	55%	45%	
Several times a year	13%	16%	
Once a month	16%	16%	
Several times a week	11%	15%	
Everyday	6%	8%	
Have you ever used MedlinePlus?	(n=291)	(n=297)	0.02
Yes	2%	6%	
No	98%	94%	
Have you ever heard of MedlinePlus?	(n=130)	(n=162)	NS
Yes	20%	29%	
No	80%	71%	
How interested are you in LEARNING how to use the Internet to find information about your health?	(n=131)	(n=164)	NS
Not interested at all	13%	13%	
Not interested	19%	18%	
Not sure	9%	13%	
Interested	31%	35%	
Very interested	28%	21%	
How USEFUL do you feel the Internet is in helping you make decisions about your health?	(n=131)	(n=164)	0.03
Not useful at all	—	1%	
Not useful	8%	2%	
Unsure	10%	16%	
Useful	46%	48%	
Very useful	36%	33%	
How IMPORTANT is it for you to be able to access health resources on the Internet?	(n=130)	(n=163)	0.02
Not important at all	—	—	
Not important	9%	4%	
Unsure	7%	16%	
Important	39%	44%	
Very important	46%	36%	

Over a three-month period (starting from the day of refurbishment), the waiting room staff at the FQHC kept track of the number of patients using the computer kiosks. Less than twenty patients used the computer kiosks in the waiting rooms, despite the refurbishment. During this time period, a part-time student working at the FQHC asked patients about barriers, if any, to using the kiosks. The most common reasons for not using the kiosks were already having access to a web-enabled smartphone, lack of interest in computers, and patients’ fear of missing when they were called for their doctor’s appointments.

#### Goal 2: Improve patients’ knowledge, attitude, and skills related to accessing online health information

Patient interest in general computer literacy classes was low. After 1 year of attempts and only being able to host 3 classes (with declining numbers of sign-ups and patient attendance over time), the idea was dropped. Therefore, the steering committee decided to conduct computer skills training in the context of a specific health condition that was relevant to patient participants (i.e., diabetes). These disease-specific training sessions had greater retention: patient attendance was 100% for 4 out of 6 of the sessions.

A review of meeting minutes revealed direct one-on-one patient engagement with MedlinePlus in the waiting room was successful but required dedicated staff or student time. Around 25% of patients were interested in these brief trainings. Patients with web experience were comfortable bookmarking the MedlinePlus site on their phones and using it in lieu of Google searches or commercially supported patient education sites. The MedlinePlus videos that were shown in recurring loops on the waiting room television monitors generated consistent attention from patients and still continue to play.

#### Goal 3: Improve clinicians’ use of MedlinePlus at the point of care

Our final goal was generally successful. Most (80%) of the clinicians and nurses at the FQHC attended 1 of the luncheon training sessions. Observational notes showed roughly half the patients agreed to brief MedlinePlus training when they were approached by clinicians or nurses in the exam room during their visits. Comparison of pre- and post-intervention surveys of clinicians and nurses (supplemental Table 4) showed a significant improvement in the proportion who recommended MedlinePlus to patients from 21% to 43% (χ^2^(1)=5.96, *p*<0.0001) (Table 5).

**Table 5:** Clinicians and nurses pre- and post-intervention survey results

	Pre-intervention (n=57)	Post-intervention (n=55)	
Have you ever visited a patient education website called MedlinePlus?			NS
Yes	70%	71%	
Have you ever recommended to a patient that they use MedlinePlus?			*p*<0.0001
Yes	21%	43%	
How often do you recommend the MedlinePlus site for patients?			NS
Once a year or less	—	4%	
Several times a year	46%	44%	
Monthly	9%	22%	
Several times a month	36%	22%	
Weekly or more	9%	9%	

However, the health center’s EHR could not accommodate MedlinePlus Connect, which would have improved MedlinePlus’s point-of-care usability. To address this challenge, we created MedlinePlus desktop icons and shortcuts to facilitate use by clinicians and nurses. We also integrated MedlinePlus into the health center EHR by creating order sets that included links to MedlinePlus. The order sets were EHR templates that provided support in making clinical decisions for medical conditions. For example, we embedded web links to relevant sections of MedlinePlus in the order set that clinicians could use when they were educating patients about hypertension.

Our process evaluation revealed patient interest at the point of care (i.e., in the exam room after their visits) was generally more favorable than in the waiting room. Our review of qualitative data indicated that the biggest barriers to integrating promotion of MedlinePlus during the AVSs was nurses’ time and space constraints, which varied by day. Specifically, during busy sessions, MedlinePlus promotion tended to slow down work flow due to the nurses’ need to screen the next patient or show the next patient to an exam room. The other challenge was staff turnover and the need for retraining in use of MedlinePlus.

## DISCUSSION

The emerging science on implementation underscores that implementation is often challenging. Some estimates indicate that two-thirds of organizations’ attempts to implement change fail [25]. Thus, it was not surprising that implementation of MedlinePlus at a FQHC met with modest success. Specifically, we found that a multimodal approach (i.e., provider training, one-on-one patient training, group patient training, and waiting room demonstrations) to embed MedlinePlus promotion and training using multiple venues improved clinicians’ and nurses’ recommendations for patients to use MedlinePlus. More importantly, we documented an improvement in the population-level usage of MedlinePlus by patients. Nevertheless, the absolute increase in reported use of MedlinePlus by patients was low.

Our results have important implications for medical librarians who are interested in promoting access to high-quality medical information among underserved patients. The first lesson is that implementation is a complex process that is affected by factors related to (1) what is being implemented, (2) the external environment of the practice, and (3) practice motivation and capability [25]. In our case, many of these factors favored implementation. Our medical librarian was experienced in working with primary care practices and effective in conducting training. The FQHC leadership believed that promotion of high-quality information was part of the organization’s mission. The clinicians and nurses were receptive to the use of MedlinePlus. We adopted a recommended approach that engaged key stakeholders in the implementation process [26]. This engagement not only helped to energize the process, but also ensured that we employed “real world” strategies that clinicians and staff viewed as feasible.

Based on our experience, we envision several roles for medical librarians who are interested in liaising with FQHCs and other organizations that provide health care for underserved patients. The first is training and teaching. Our medical librarian was invaluable in assisting in development of training materials for conducting clinician and nurse trainings. In addition, as medical librarians are important resources for patients, it may be helpful to include medical librarians in group visits for patients. Often group leaders invite outside experts to join the group as guests for a dedicated session on a particular topic [27]. Medical librarians can serve an important consultative role for practices that are interested in promoting educational materials and resources, such as MedlinePlus, to their patients.

Our findings come with caveats. The findings are based on work at a single large FQHC and might not necessarily generalize to all FQHCs nationally. We used an iterative, multicomponent approach that sought to embed favorable knowledge, attitudes, and skills related to MedlinePlus in as many venues as possible. We cannot determine which component contributed the most to improvements. We conducted cross-sectional analyses using data from clinicians and staff as well as patients in the waiting room. These methods are associated with various sampling biases and temporal changes in the samples over time. Thus, we cannot be sure that improvements resulted from our strategies. However, we are not aware of any MedlinePlus promotion, event, or news feature during this time window beyond our interventions that could have affected clinician use of MedlinePlus.

We conclude that integrating MedlinePlus at multiple levels and into multiple care processes provides a potential means for promoting its use among underserved patients. Further work is needed to devise even more effective and efficient means for promoting MedlinePlus in FQHCs.

## SUPPLEMENTAL FILES

Appendix A Patient survey

Appendix B Clinician and nurse survey

Table 2 Patient pre- and post-intervention survey results: demographics

Table 4 Clinicians and nurse pre- and post-intervention survey results
