# Supplementary material for: Promoting MedlinePlus utilization in a federally qualified health center using a multimodal approach
Source: J Med Libr Assoc. 2018 Jul 1;106(3):361–9. doi: 10.5195/jmla.2018.216 (PMC6013131; doi:10.5195/jmla.2018.216)
Supplement: Appendix A [file jmla-106-361-s001.pdf]

JMLA Journal of the Medical Library Association

SC

Supplemental content to
J Med Libr Assoc. Jul;106(3):dx.doi.org/10.5195/jmla.2018.216
www.jmla.mlanet.org
© Sanders, Bringley, Thomas, Boyd, Farah, Fiscella 2018

Promoting MedlinePlus utilization in a federally qualified health center using a multimodal approach

Mechelle Sanders, BA; Kate Bringley, BS; Marie Thomas; Michele Boyd, MPA; Subrina Farah, MS; Kevin Fiscella, MD, MPH

APPENDIX A

Patient survey

1. What is your age? __ years

2. What is your gender?
__ Male
__ Female
__ Transgendered

3. What is your race or ethnicity?
__ Asian
__ Black
__ Hispanic
__ White
__ Other/mixed

4. What is your education degree?
__ No high school degree or GED
__ High school or GED only
__ Associate college degree
__ College degree
__ Master's or higher degree

5. What is your preferred reading language?
__ Cannot read at all
__ English
__ Spanish
__ Other______

6. How often do you use the Internet?
__ Never
__ Several times a year
__ Once a month
__ Several times a week
__ Every day

7. How often have you used the Internet to *look up information about your health?*
__ Never [skip questions 9–15]
__ Several times a year
__ Once a month
__ Several times a week
__ Every day

SC
Supplemental content to
J Med Libr Assoc. Jul;106(3):dx.doi.org/10.5195/jmla.2018.216
www.jmla.mlanet.org
© Sanders, Bringley, Thomas, Boyd, Farah, Fiscella 2018

8. How interested are *you in learning how* to use the Internet to find information about your health?
__ Not interested at all
__ Not interested
__ Not sure
__ Interested
__ Very interested

9. Have you heard of MedlinePlus?
__ Yes
__ No [skip questions 13–14]

10. How USEFUL do you feel the Internet is in helping you make decisions about your health?
__ Not useful at all
__ Not useful
__ Unsure
__ Useful
__ Very useful

11. How IMPORTANT is it for you to be able to access health resources on the Internet?
__ Not important at all
__ Not important
__ Unsure
__ Important
__ Very important

12. Have you EVER USED MedlinePlus?
__ Yes
__ No (skip questions 13–14]

13. How EASY was it to find the information you wanted with MedlinePlus?
__ Very difficult
__ Difficult
__ So-so
__ Easy
__ Very easy

14. How LIKELY are you to go online and use MedlinePlus in the future?
__ Very likely
__ Likely
__ Not sure
__ Unlikely
__ Very unlikely

15. How interested would you be in attending a training session in how to use the Internet to find information and use your portal to look at your test results and make appointments?
__ Not interested at all
__ Not interested
__ Not sure
__ Interested
__ Very interested

Supplemental content to
J Med Libr Assoc. Jul;106(3):dx.doi.org/10.5195/jmla.2018.216
www.jmla.mlanet.org
© Sanders, Bringley, Thomas, Boyd, Farah, Fiscella 2018

16. Do you own or have easy access to a computer?
__ Yes, I have one at home
__ Yes, I use the computer at the library
__ Yes, I use a computer at a friend or relative's house
__ No I don't have a computer and don't have access to one
__ I have a computer or access to one but don't know how to use it
__ I have a computer or access to one but don't want to use it

17. Do you use a smartphone (iPhone, Android) for Internet or apps?
__ Never
__ Several times a year
__ Once a month
__ Several times a week
__ Every day

18. Did you know you can request appointments, request medication refills, and view your test results using the Internet by signing up for the health center portal?
__ Yes
__ No [skip question 19]

19. How easy was the portal to use?
__ Very difficult
__ Difficult
__ So-so
__ Easy
__ Very easy

20. How interested would are you be in using the portal if someone showed you how to use it?
__ Not interested at all
__ Not interested
__ Not sure
__ Interested
__ Very interested

21. How interested would you be in using the portal if you could use it as an app on your smartphone?
__ Not interested at all
__ Not interested
__ Not sure
__ Interested
__ Very interested
