# Supplementary material for: Promoting MedlinePlus utilization in a federally qualified health center using a multimodal approach
Source: J Med Libr Assoc. 2018 Jul 1;106(3):361–9. doi: 10.5195/jmla.2018.216 (PMC6013131; doi:10.5195/jmla.2018.216)
Supplement: Appendix B [file jmla-106-361-s002.pdf]

Journal of the Medical Library Association

Supplemental content to
J Med Libr Assoc. Jul;106(3):dx.doi.org/10.5195/jmla.2018.216
www.jmla.mlanet.org
© Sanders, Bringley, Thomas, Boyd, Farah, Fiscella 2018

Promoting MedlinePlus utilization in a federally qualified health center using a multimodal approach

Mechelle Sanders, BA; Kate Bringley, BS; Marie Thomas; Michele Boyd, MPA; Subrina Farah, MS; Kevin Fiscella, MD, MPH

APPENDIX B

Clinician and nurse survey

1. Name of the site at which you mostly work: ________________

2. Position type:
__ Licensed practical nurse (LPN)
__ MA
__ Nurse practitioner
__ Physician
__ Physician assistant
__ Registered nurse (RN)
__ Resident

3. Gender:
__ Male
__ Female
__ Other

4. Length of time with the health center:
__ Less than 1 year
__ 1–5 years
__ More than 5 years

5. Have you ever visited a patient education site called MedlinePlus?
__ Yes
__ No

6. Have you ever recommended to a patient that they use MedlinePlus?
__ Yes
__ No

7. How often do you recommend the MedlinePlus site to patients?
__ Weekly or more
__ Several times a month
__ Monthly
__ Several times a year
__ Once a year or less
