# Supplementary material for: Promoting MedlinePlus utilization in a federally qualified health center using a multimodal approach
Source: J Med Libr Assoc. 2018 Jul 1;106(3):361–9. doi: 10.5195/jmla.2018.216 (PMC6013131; doi:10.5195/jmla.2018.216)
Supplement: Table 2 [file jmla-106-331-t002.pdf]

JMLA Journal of the Medical Library Association

SC
Supplemental content to
J Med Libr Assoc. Jul;106(3):dx.doi.org/10.5195/jmla.2018.216
www.jmla.mlanet.org
© Sanders, Bringley, Thomas, Boyd, Farah, Fiscella 2018

Promoting MedlinePlus utilization in a federally qualified health center using a multimodal approach

Mechelle Sanders, BA; Kate Bringley, BS; Marie Thomas; Michele Boyd, MPA; Subrina Farah, MS; Kevin Fiscella, MD, MPH

Table 2

Patient pre- and post-intervention survey results: demographics

	Pre-intervention (n=302)	Post-intervention (n=302)	
Age (mean)	46	45	NS
Gender (n)			0.04
Male	34%	43%	
Female	66%	57%	
Transgender	<1%	–	
Race/Ethnicity			NS
Asian	<1%	3%	
Black	67%	66%	
White	10%	11%	
Other/Mixed	6%	8%	
Hispanic	16%	13%	
Education			NS
Less than 9th grade	7%	6%	
9th–11th grade	25%	29%	
High school/GED	36%	33%	
Some college	21%	24%	
College grad or more	11%	6%	
Prefer not to answer	–	1%	
Don't know	<1%	<1%	
Preferred reading language			NS
English	88%	88%	
Spanish	10%	8%	
Other	2%	3%	
Cannot read at all	–	1%	
Access to a computer			NS
Home	53%	60%	
Library	9%	7%	
Friend/relative	9%	7%	
No access	28%	25%	
Access, don't know how to use it	2%	1%	
Access, don't want to use it	1%	<1%	

JMLA Journal of the Medical Library Association

Supplemental content to
J Med Libr Assoc. Jul;106(3):dx.doi.org/10.5195/jmla.2018.216
www.jmla.mlanet.org
© Sanders, Bringley, Thomas, Boyd, Farah, Fiscella 2018

	**Pre-intervention (n=302)**	**Post-intervention (n=302)**	
Internet use via smartphone			0.02
Never	47%	38%	
Several times a year	1%	<1%	
Once a month	2%	1%	
Several times a week	6%	4%	
Everyday	44%	57%	
Internet use			NS
Never	30%	24%	
Several times a year	4%	6%	
Once a month	7%	5%	
Several times a week	15%	15%	
Everyday	44%	50%	
