# Supplementary material for: Promoting MedlinePlus utilization in a federally qualified health center using a multimodal approach
Source: J Med Libr Assoc. 2018 Jul 1;106(3):361–9. doi: 10.5195/jmla.2018.216 (PMC6013131; doi:10.5195/jmla.2018.216)
Supplement: Table 4 [file jmla-106-331-t004.pdf]

JMLA Journal of the Medical Library Association

SC

Supplemental content to
J Med Libr Assoc. Jul;106(3):dx.doi.org/10.5195/jmla.2018.216
www.jmla.mlanet.org
© Sanders, Bringley, Thomas, Boyd, Farah, Fiscella 2018

Promoting MedlinePlus utilization in a federally qualified health center using a multimodal approach

Mechelle Sanders, BA; Kate Bringley, BS; Marie Thomas; Michele Boyd, MPA; Subrina Farah, MS; Kevin Fiscella, MD, MPH

Table 4

Clinicians and nurse pre- and post-intervention survey results

	Baseline (n=57)	Post intervention (n=55)	*p*-value
Center			NS
1	39%	33%	
2	35%	28%	
3	2%	23%	
4	24%	38%	
Position			NS
Licensed practical nurse (LPN)	27%	49%	
MA	–	2%	
Nurse practitioner	20%	19%	
Physician	35%	19%	
Physician assistant	6%	3%	
Resident	8%	3%	
Registered nurse (RN)	4%	5%	
Gender			NS
Female	86%	89%	
Length of time at Anthony L. Jordan Health Center			NS
Less than 1 year	20%	20%	
